# Clinical features of three patients with paradoxical immune reconstitution inflammatory syndrome associated with *Talaromyces marneffei* infection

**DOI:** 10.1016/j.mmcr.2016.12.005

**Published:** 2016-12-09

**Authors:** Nguyen Tat Thanh, Le Duc Vinh, Nguyen Thanh Liem, Cecilia Shikuma, Jeremy N. Day, Guy Thwaites, Thuy Le

**Affiliations:** a Oxford University Clinical Research Unit, 764 Vo Van Kiet, Quan 5, Ho Chi Minh City, Vietnam; b Vietnam Hospital for Tropical Diseases, 764 Vo Van Kiet, Quan 5, Ho Chi Minh City, Vietnam; c Centre for Tropical Medicine and Global Health, University of Oxford, Old Road Campus, Roosevelt Drive, Oxford OX3 7FZ, UK; d Hawaii Center for AIDS, University of Hawaii at Manoa, 651 Ilalo St., BSB, Suite 231, Honolulu, HI 96813, USA

**Keywords:** Talaromycosis, Penicilliosis, Talaromyces marneffei, Penicillium marneffei, Immune reconstitution inflammatory syndrome, HIV

## Abstract

*Talaromyces marneffei* infection is a major cause of death in HIV-infected individuals in South and Southeast Asia. Talaromycosis immune reconstitution inflammatory syndrome has not been well described. Here we report the clinical features, management, and outcomes of three HIV-infected patients with talaromycosis-associated paradoxical immune reconstitution inflammatory syndrome in Ho Chi Minh City, Vietnam.

## 1 Introduction

Talaromycosis (formerly penicilliosis) is a systemic mycosis caused by the dimorphic fungus *Talaromyces* (formerly *Penicillium*) *marneffei* and is amongst the most common HIV-associated opportunistic infections in south and southeast Asia, alongside tuberculosis, cryptococcal meningitis and *Pneumocystis jiroveci* pneumonia [1], [2]. Talaromycosis occurs in patients with advanced HIV infection, generally when the CD4+ T-cell count is less than 100 cells/µL [3]. In severely immunosuppressed patients, the institution of antiretroviral therapy (ART) is associated with a high risk of the immune complication termed immune reconstitution inflammatory syndrome (IRIS). This syndrome is a result of exaggerated or dysregulated T-cell responses to either viable pathogen or persistent pathogen derived antigens, and is driven by the institution of ART. IRIS manifests in one of two forms: a ‘paradoxical’ worsening of a treated or under treatment opportunistic infection (paradoxical IRIS) or the uncovering of previously ‘occult’ or subclinical infections (unmasking IRIS) [4]. The incidence of IRIS ranges from 10% to 40% and varies by pathogen, being most commonly described in patients with infections caused by mycobacteria (particularly tuberculosis), fungi (particularly cryptococcal meningitis), and lymphotropic viruses such as JC virus (the cause of progressive multifocal leukoencephalopathy) and human herpes viruses [4], [5]. To date there have been few case reports of IRIS associated with *T. marneffei* infection [6], [7], [8], [9], [10], [11], [12], [13], and the incidence and risk factors have not been defined. All reported cases were classified as unmasking IRIS [7], [8], [9], [10], [12], [13], except for one pediatric case from India that was classified as paradoxical IRIS [6]. Here we described three adult patients with talaromycosis-associated paradoxical IRIS at the Hospital for Tropical Diseases in Ho Chi Minh City in order to add to the currently sparse knowledge on the clinical characteristics and management of these patients.

## 2 Cases

**Fig. 1:**
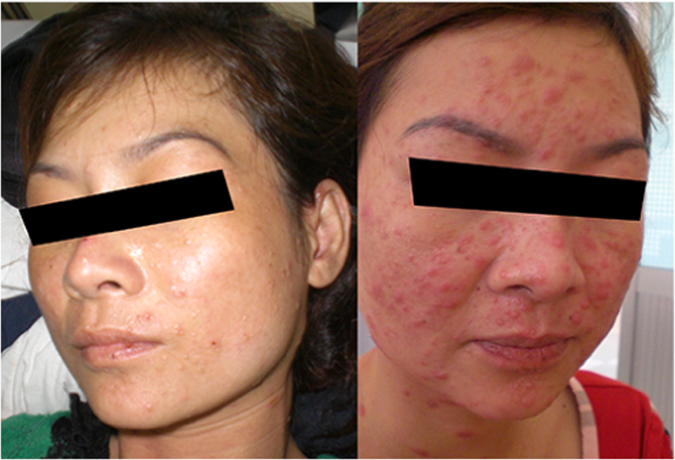
The characteristic central necrotic skin lesions in patient 1 at time of talaromycosis (left) and the erythematous dermatitis-like skin lesions of the same patient at the time of talaromycosis IRIS (right).

**Patient 1:** A 31 year-old female from the Central Highland region north of Ho Chi Minh City presented in October 2013 with a history of 4 weeks of fever, 5 kg of weight loss, throat pain, vomiting, and fatigue. The development of skin nodules on her face and body prompted her to seek care at our hospital, where she was diagnosed with HIV infection by three different enzyme-linked immunosorbent assay (ELISA), and with talaromycosis by skin and blood culture. She had no history of injection drug use (IDU). The CD4+ T-cell count was 10 cells/µL. Her laboratory tests revealed pancytopenia and elevated aspartate aminotransferase (AST) (Table 1). She was treated with itraconazole 400 mg/day with complete resolution of fever and skin lesions by D+11; however hemoculture performed on D+14 remained positive. She began ART with the national first-line ART regimen that included tenofovir, lamivudine, and efavirenz on D+39. She was feeling well and was gaining weight until month 5 on ART, 170 days after her initial diagnosis, when her clinical status abruptly changed. She developed high fever (body temperature 39.5 °C), new skin lesions in the same distribution as before but which were significantly more erythematous (Fig. 1), enlargement of cervical lymph nodes, and interphalangeal joint pain, tenderness and swelling in both hands. She reported good adherence with her ART and itraconazole secondary prophylaxis. She was readmitted to hospital and received empirical antibiotic therapy with intravenous ceftriaxone, intravenous fluid infusion, and ibuprofen for joint pain for 10 days. The CD4+T-cell count on this admission was 100 cells/µL. The full blood count and biochemistry tests were in normal ranges. Microscopy and culture of skin lesions along with hemoculture and fine needle aspiration (FNA) of a neck lymph node were negative for *T. marneffei* and other organisms. Multiple acid fast bacilli (AFB) smears of sputum and lymph node were negative, and chest x-ray showed no abnormality. The diagnosis of talaromycosis IRIS was made based on the temporal association between the timing of ART, the appearance of the inflamed skin lesions and joints, and the significant rise in CD4+ count. The dose of itraconazole was increased from 200 mg/day to 400 mg/day for 12 further weeks, and ART was continued. She was discharged after 2 weeks and continued to improve on ART. She received a total 23 months of itraconazole therapy. At her last follow up visit in August 2015, she was well, and her CD4+ T-cell count was 192 cells/µL.

**Table 1:** Clinical features, treatment, and outcomes of three HIV-infected patients with talaromycosis-associated paradoxical immune reconstitution inflammatory syndrome in Ho Chi Minh City, Vietnam.

**Case**	**Age and sex**	**IDU**	**Status at*****T. marneffei*****diagnosis prior to ART initiation**		**Microbiology at time of*****T. marneffei*****diagnosis**	**Time to IRIS onset**	**Status at IRIS presentation**		**Microbiology of at time of IRIS**	**Treatment**	**Response**
			**Clinical and laboratory features**	**CD4 count**			**Clinical and laboratory features**	**CD4 count**			
1	32 year-old female	No	Fever, central necrotic skin lesions, oral ulcer, and hepatosplenomegaly Hemoglobin=9 g/dL Platelet=88 K/µL AST =193 U/L ALT = 45 U/L	09 (cells/µL)	Skin microscopy and culture positive Hemoculture positive	5 months	Fever, dense erythematous skin lesions, lymphadenopathy, bilateral interphalangeal joint swelling and pain Hemoglobin=11.2 g/dL Platelet=306 K/µL AST=46 U/L ALT=27 U/L	100 (cells/µL)	Skin microscopy and culture negative Hemoculture negative	Itraconazole 400 mg/day for 12 weeks NSAIDs for 10 days	Complete resolution of symptoms after one month
2	34 Year-old male	Yes	Fever and skin lesions Laboratory values unavailable	09(cells/µL)	Skin microscopy and culture positive Hemoculture was not done	3months	No fever, psoriasis-like papules, and lymphadenopathy Hemoglobin=10. 9 g/dL Platelet=225 K/µL AST=58 U/L ALT=69 U/L	94 (cells/µL)	Skin microscopy and culture positive Hemoculture negative	Amphotericin B 0.7 mg/kg/day for 2 weeks then itraconazole 400 mg/day for 10 weeks NSAIDs for 5 days	Complete resolution of symptoms after one month
3	25 year-old female	No	Fever, sporadic central necrotic skin lesions, vomiting, and wasting syndrome Hemoglobin=5.8 g/dL Platelet=100 K/µL	02(cells/µL)	Skin microscopy and culture positive Hemoculture was not done	5 months	Fever, new central necrotic skin lesions, multiple purulent ulcers in both legs, bilateral interphalangeal joint swelling and pain, and large erythema nodosum lesion in the right arm Hemoglobin=11.2 g/dL Platelet count=355 K/µL AST=24 U/L ALT=23 U/L	96 (cells/µL)	Skin microscopy negative Skin culture positive Hemoculture negative AFB smear positive in sputum and skin ulcers	Amphotericin B, 0.7 mg/kg/day for 2 weeks then itraconazole 400 mg/day for 10 weeks NSAIDs for 5 days Anti-tuberculous therapy: isoniazid, rifampicin, ethambutol, pyrazinamide	Gradual clinical improvement over three months

Note: IDU, intravenous drug user; IRIS, immune reconstitution inflammatory syndrome; ART, antiretroviral therapy; AFB, acid fast bacilli; NSAIDs, nonsteroidal anti-inflammatory drugs, AST, aspartate aminotransferase; ALT, alanine transaminase; K=1,000 units

**Fig. 2:**
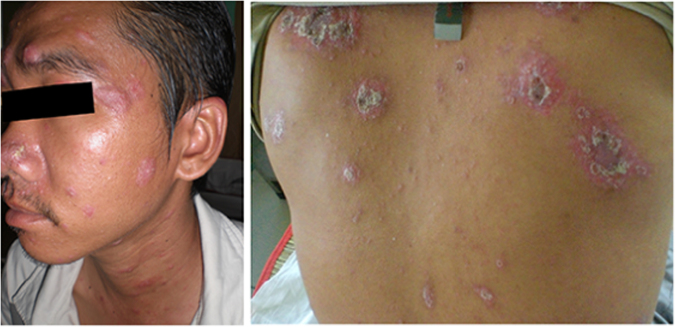
The atypical psoriasis-like skin lesions on the face and back in patient 2 at time of talaromycosis IRIS.

**Patient 2:** A 35 year-old male with a history of IDU from Ho Chi Minh City presented in May 2013 with a two week history of fatigue and scaly skin lesions on his face and body without fever. He had been diagnosed with HIV and *T. marneffei* infection by skin microscopy and culture at another hospital and had started itraconazole therapy (currently on a 200 mg/day maintenance dose). He was started on first-line ART 3 months prior to presentation, at which time his CD4+ T-cell count was 9 cells/µL, and reported good adherence to ART and itraconazole maintenance therapy. The skin lesions on this presentation were significantly larger and different from before and were scaly psoriasis-like papules on his face and trunk (Fig. 2). The skin microscopy revealed yeast-like organisms that had midline septa, and skin culture was positive for *T. marneffei*. Hemoculture was negative. The CD4+ T-cell count was 94 cells/µL. The dose of itraconazole was increased from 200 mg/day to 400 mg/day for suspected talaromycosis IRIS. However, no improvement of skin lesions was observed after one week. Itraconazole was therefore changed to intravenous amphotericine B 0.7 mg/kg/day with substantial improvement of skin lesions after two weeks. The patient was discharged on itraconazole 400 mg/day for further 10 weeks. He went on to receive a total of 36 months of itraconazole therapy. At the last follow up visit in February 2016, the skin lesions completed resolved, and his CD4+ T-cell count was 260 cells/µL.

**Fig. 3:**
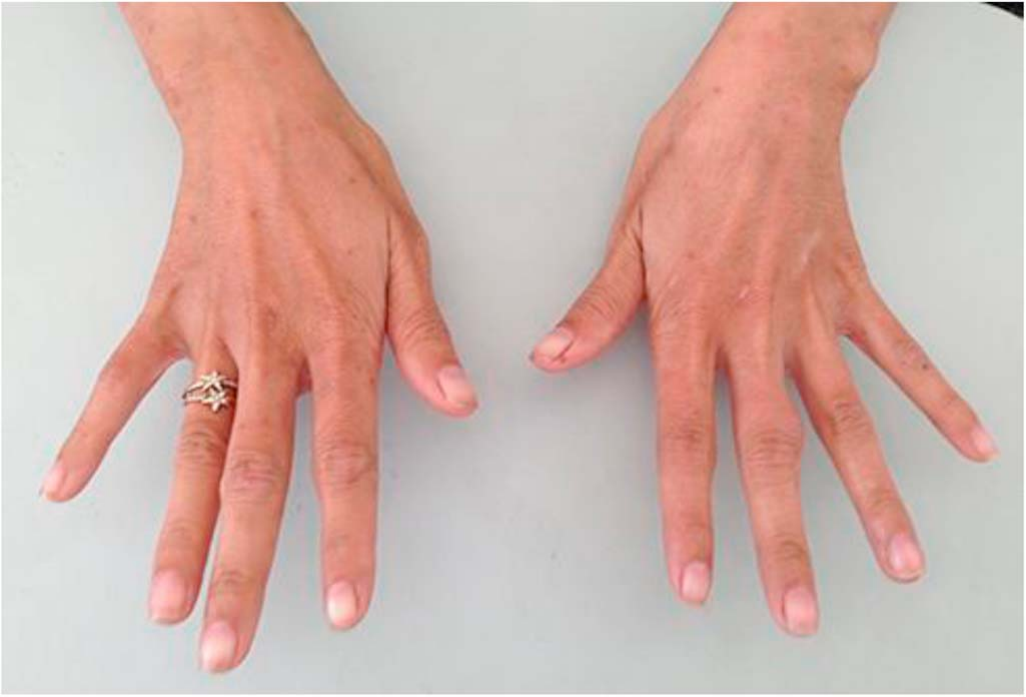
Bilateral synovitis of the proximal interphalangeal joints in the index and middle fingers of patient 3 at time of talaromycosis IRIS.

**Fig. 4:**
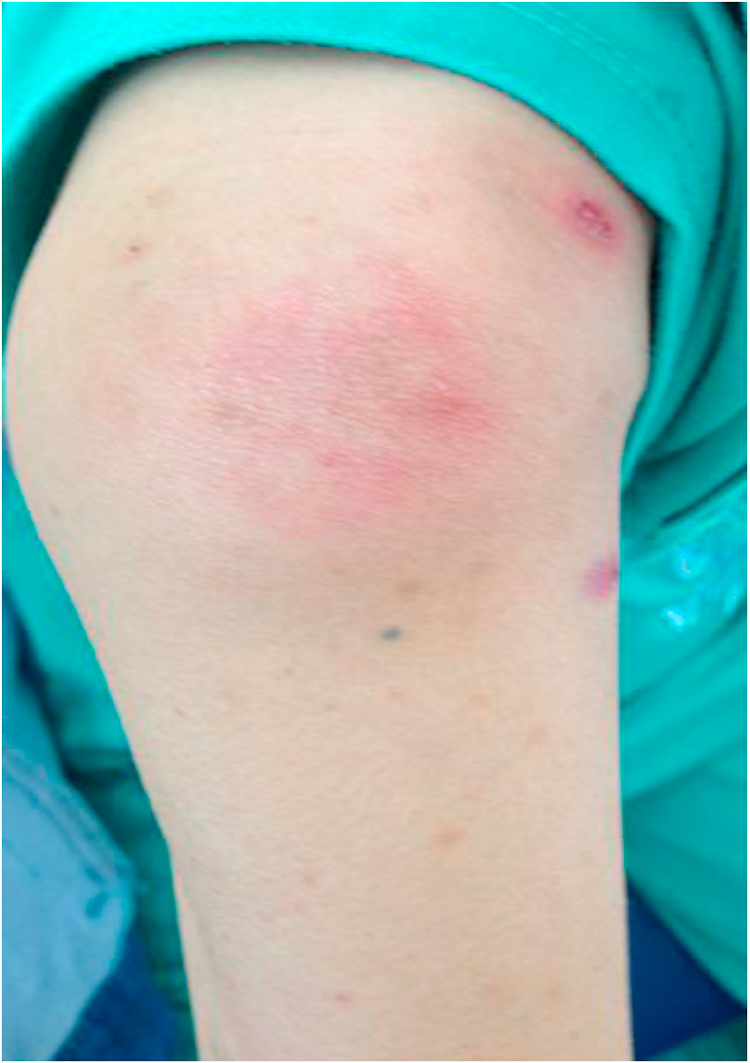
A large erythema nodosum lesion presented in left arm of patient 3 at time of talaromycosis IRIS.

**Patient 3:** A 25 year-old female from Tay Ninh province on the border of Cambodia northwest of Ho Chi Minh City presented in February 2015 with one month of fever, fatigue, cervical lymphadenopathy, and skin lesions. She was diagnosed with HIV and *T. marneffei* infection based on positive skin microcopy and culture. At presentation the CD4+ T-cell count was 02 cells/µL. She was treated with itraconazole 400 mg/day with resolution of fever and skin lesions and was discharged on D+14. She began first-line ART on D+37. She was doing well and gaining weight until month 5 on ART when she developed high fever, new purulent skin lesions on her face and legs, and bilateral interphalangeal joint swelling and pain (Fig. 3). She had a large lesion resembling erythema nodosum on the extensor surface of the right elbow (Fig. 4). Her hematology and biochemistry results were in normal ranges, and her CD4 T-cell was 96 cells/µL on D+167. Abdominal ultrasound performed for abdominal pain revealed new intra-abdominal lymphadenopathy. Chest X-ray was normal. Skin culture was positive for both *T. marneffei* and methicillin-sensitive *Staphylococcus aureus* (MSSA). Hemoculture was negative. She was treated with intravenous amphotericin B deoxycholate and intravenous oxacillin for MSSA super-infection of the skin lesions, both for 2 weeks plus one week of ibuprofen for joint pain. Her fever and skin lesions resolved after 10 days; however she developed productive cough and cervical lymph node enlargement during hospitalization. FNA of the lymph node showed no organism; however her sputum AFB smear was positive and she was started on antituberculosis therapy with isoniazid, rifampicin, ethambutol, and pyrazinamide. She was discharged after 19 days of hospitalization on antituberculosis therapy, and the dose of itraconazole was increased to the maximum dose of 600 mg/day for further 10 weeks to account for the rifampicin-induced reduction in itraconazole levels, as therapeutic monitoring for itraconazole was not available in Vietnam. The patient had a gradual improvement of symptoms over 3 months. At the last follow up visit in June 2016, she was completely well, and the CD4 T-cell count had increased to 170 cells/µL.

## 3 Discussion

The plethora or presentations of IRIS are in part driven by the underlying pathogen. Although there is no gold standard or a single test that can confirm the diagnosis, a number of pathogen specific definitions of IRIS have been proposed [5], [14], [15], [16] and can provide guidance to characterize IRIS associated with talaromycosis. Generally there must be a temporal relationship between timing of ART and subsequent development of symptoms (usually within the first 3–6 months). Most case definitions require evidence of immune restoration, either by demonstration of a decrease of plasma HIV RNA levels by more than 1 log_10_ copies/ml or an increase in CD4+ T-cell count from baseline. In resource poor areas receipt of and adherence to an effective ART regimen can be sufficient. The clinical features should be consistent with an inflammatory process, and the clinical process should not be explained by a new infectious process, by drug toxicity or drug resistance [5]. The time from ART initiation to the IRIS event in our three patients was 3–5 months. Although HIV RNA quantification is not the standard of care in Vietnam, the significant rise in CD4+ T-cell counts in all three patients provides the supportive evidence of immune restoration. All three patients had clinical signs consistent with an inflammatory process. Patient 1 developed dense erythematous skin lesions on her face resembling dermatitis (Fig. 1). She also developed new cervical lymphadenopathy and synovitis of the interphalangeal joints that were not present during the first presentation. Patient 2 had unusual psoriatic-like skin lesions that are not characteristic for talaromycosis (Fig. 2). These lesions were much larger in size, and the scaling feature was new according to the patient. Patient 3 developed bilateral synovitis of the interphalangeal joints (Fig. 3) and had a large erythema nodosum lesion on her arm (Fig. 4), although erythema nodosum could also be a manifestation of tuberculosis IRIS in this patient. Similar to our three patients, signs of an inflammatory process were also present in a 12 year-old patient reported from India with paradoxical IRIS. He was described as having exaggerated skin lesions compared to his initial presentation (although no photo was available) along with a severe inflammatory arthritis [6]. This patient had achieved an unusually rapid CD4+ T-cell recovery from 11 to 172 cells/µL over only a single month on ART. With respect to laboratory features, it is notable that hemoglobin, platelet, and liver transaminase levels returned close to normal ranges in all three patients at the time of IRIS. These laboratory characteristics, in addition to a rise in CD4+ T-cell counts, might aid the differentiation of patients with IRIS and those with disease relapse, as culture can be positive in both conditions. Unlike paradoxical IRIS, significant inflammation does not appear to be a prominent feature in the reported cases of unmasking IRIS in the literature, where patients tended to present with features similar to disseminated talaromycosis (skin lesions, lymphadenopathy, hepatosplenomegaly, and anemia) [7], [8], [9], [10], [11], [12], [13].

All three of our patients required hospitalization for exclusion of other active infections, re-treatment with high dose antifungals, and supportive care. All patients recovered with continuation of ART. Although talaromycosis IRIS can be clinically challenging and can have significant morbidity, the outcomes of our patients and of those reported in the literature with unmasking talaromycosis IRIS appear to be better than seen in IRIS associated with cryptococcal meningitis, where mortality is as high as 34% [17]. Non-steroidal anti-inflammatory treatment was useful in two of our patients, and seemed adequate for the control of inflammatory synovitis without the need for corticosteroid treatment, although corticosteroid may be required for patients with more severe symptoms. A previous case from China reports the use of methyl prednisolone in a patient with unmasking IRIS [13]. It is unknown whether intensification of antifungal therapy with amphotericin B or increased dose of itraconazole is required in patients with culture-negative paradoxical IRIS, such as patient 1. However it is generally believed that residual pathogen, viable or not, plays an important role in the pathogenesis of IRIS [5]. Randomized trials to define optimal anti-infective treatment for IRIS associated with invasive fungal infections have not been performed.

Co-infection with talaromycosis and tuberculosis is common, with microbiology-confirmed tuberculosis coinfection seen in 22% of patients [3], and presents a therapeutic challenge in that rifampicin as a potent CYP3A4 inducer reduces itraconazole concentration by 60% [18]. Co-administration of itraconazole and rifampicin might increase the risk for talaromycosis relapse or IRIS in co-infected patients; however the clinical significance of this important pharmacokinetic interaction has not been systematically investigated. Patient 3 in addition to having paradoxical IRIS associated with talaromycosis also developed unmasking IRIS associated with tuberculosis requiring rifampicin therapy. She was retreated with amphotericin B and avoided this important drug-drug interaction during the initial period of therapy. She tolerated the increased dose of itraconazole to 600 mg/day during rifampicin co-administration, and had no disease relapse. Co-administration of itraconazole and efavirenz also presents a pharmacokinetic challenge in that efavirenz is a moderate inducer of CYP3A4 and reduces the level of itraconazole by 30% when the two drugs are co-administered [11], [19]. However, the clinical significance of this pharmacokinetic interaction is not apparent as the majority of HIV-infected patients with talaromycosis in Asia are safely started on ART regimen containing efavirenz with low rates of disease relapse when they are maintained on secondary itraconazole prophylaxis [20].

This case study highlights the clinical manifestations and management in patients with paradoxical talaromycosis IRIS and call for research to define the incidence, risk factors, and clinical impact of this understudied complication in patients with advanced HIV infection in Asia.

## Conflict of Interest

We declare that there are no competing interests.
